# Bamboo shoot dietary fiber alleviates gut microbiota dysbiosis and modulates liver fatty acid metabolism in mice with high-fat diet-induced obesity

**DOI:** 10.3389/fnut.2023.1161698

**Published:** 2023-03-09

**Authors:** Xiaolu Zhou, Lingjun Ma, Li Dong, Daotong Li, Fang Chen, Xiaosong Hu

**Affiliations:** Key Laboratory of Fruits and Vegetables Processing, College of Food Science and Nutritional Engineering, National Engineering Research Centre for Fruits and Vegetables Processing, Ministry of Agriculture, Engineering Research Centre for Fruits and Vegetables Processing, Ministry of Education, China Agricultural University, Beijing, China

**Keywords:** bamboo shoot dietary fiber, obesity, insulin resistance, gut microbiota, metabolites, antibiotic-treated mice

## Abstract

**Introduction:**

Obesity is a common nutritional disorder characterized by an excessive fat accumulation. In view of the critical role of gut microbiota in the development of obesity and metabolic diseases, novel dietary therapies have been developed to manage obesity by targeting the gut microbiome. In this study, we investigated anti-obesity effects of bamboo shoot dietary fiber (BSDF) and the potential mechanisms.

**Methods:**

After 12 weeks of intervention with BSDF in high-fat mice, we detected obesity-related phenotypic indicators, and made transcriptomic analysis of liver tissue. Then we analyzed the changes of gut microbiota using 16S rRNA gene sequencing, explored the effect of BSDF on gut microbiota metabolites, and finally verified the importance of gut microbiota through antibiotic animal model.

**Results and discussion:**

We found that BSDF was effective in reducing lipid accumulation in liver and adipose tissue and alleviating dyslipidemia and insulin resistance. Liver transcriptome analysis results showed that BSDF could improve lipid metabolism and liver injury by modulating peroxisome proliferator-activated receptor (PPAR) and fatty acid metabolic pathways. The 16S rRNA gene sequencing analysis of gut microbiota composition showed that BSDF significantly enriched beneficial bacteria such as *Bifidobacterium, Akkermansia, Dubosiella*, and *Alloprevotella*. Analysis of fecal metabolomics and gut microbiota metabolites revealed that BSDF increased the levels of several short-chain fatty acids and enriched bile acids, which may be important for improving lipid metabolism. Notably, the obesity-related metabolic disorders were abrogated after the abrogation of gut microbiota, suggesting that gut microbiota is a key factor in the beneficial effects of BSDF.

**Conclusion:**

Our study suggests that BSDF as a prebiotic supplement has the potential to improve obesity by improving gut microbiota and modulating host PPAR and fatty acid metabolic pathways.

## 1. Introduction

Obesity has become a global epidemic, whose essence is caused by the imbalance between energy intake and consumption (1), which is closely related to hypertension, hyperlipidemia, type 2 diabetes and alcoholic fatty liver. It is urgent to vigorously develop drugs and methods to treat obesity. However, at present, obesity drugs and surgical treatment methods not only bring huge economic burden, but also some side effects (2, 3). Therefore, it is particularly important to treat obesity safely and efficiently through scientific and reasonable lifestyle changes (especially dietary patterns).

The development of obesity is associated with a number of factors, of which the gut microbiota is one of the key factors that has received a lot of attention in recent years and is a potential target for the prevention of obesity and related metabolic diseases. And nutrients and active substances in food can effectively regulate the gut microbiota (4–6), among which insoluble dietary fiber has a good effect on regulating the structure and function of the gut microbiota (7), but there is less research on obesity reduction and its mechanism of action (8, 9). At the same time, the structure and function of different sources of dietary fiber vary greatly, and its effect on obesity and its mechanism of action are still unclear. To explore this mechanism, Qiong bamboo shoots (BS) containing abundant dietary fiber were used as study subjects, which is the young shoots of the *Qiongzhuea tumidinoda*, and a small to medium-sized bamboo mainly distributed in Sichuan and Yunnan provinces in China. Qiong bamboo shoot dietary fiber (BSDF) is an dietary fiber extracted from the buds of BS, which has a higher yield of insoluble dietary fiber than other plants (10). Previous studies have shown that dietary fiber, as an important energy source of gut microbiota, has beneficial effects on gut metabolites, and can produce SCFAs and other metabolites under the condition of anaerobic bacteria decomposition (11, 12). In addition, metabolites of dietary fiber gut, including short-chain fatty acids (SCFAs), are also known to have various beneficial effects on host physiology (13). Given the benefits of dietary fiber in improving gut homeostasis, we hypothesize that gut microbiota may play an irreplaceable role in the beneficial effects of BSDF on metabolic disorders.

In order to verify our hypothesis, this study first tested the relevant phenotypes (body weight, fat weight, insulin resistance, etc.) after the intervention of BSDF in obesity. The phenotypic changes and molecular mechanisms were preliminarily verified by liver transcriptome and Real-Time Quantitative PCR (RT-qPCR) experiments. Next, focusing on gut microorganisms, we explored the effect of BSDE on gut microbiota through 16S rNA, and determined gut metabolites through fecal metabolomics, so as to further explore the underlying mechanism of BSDF. Finally, we used antibiotics to remove most of the bacteria in mice to verify the necessity of the existence of gut microbiota. With these results, we provide new insights into the mechanisms by which BSDF improves obesity through the gut microbiota and promote a more comprehensive understanding of the relationship between BSDF and the gut microbiota.

## 2. Materials and methods

### 2.1. Preparation of BSDF

The BSDF extraction refers to the method of Bangoura et al. with appropriate modifications (12). Freeze-dried powder of Bamboo shoots was provided by Shan Yibao Biotechnology Co., Ltd. (Yunnan Yiliang China) and pulverized into powder (80-mesh). BSDF was extracted by enzymatic hydrolysis. Briefly, distilled water (200 mL) was added to the weighed bamboo shoot freeze-dried powder (5 g), and magnetically stirred for 15 min (Bodajingke Instrument Co., Ltd., Shenzhen, China). The temperature was kept constantly at 40°C. After adjusting the pH to 7.0, 5000 U/mL neutral protease (Solarbio Biotechnology Co., Ltd., Beijing, China) was added, and further stirred for 120 min. Then, the mixture was filtered through a 400-mesh filter cloth, obtained residue was washed three times with distilled water at 40°C. Finally, washed residue was freeze-dried at a vacuum freeze dryer (Vaco 5 ZIRBUS, Beijing Hanmei Biotechnology Co., Ltd., Beijing, China), and sieved (100 mesh). The prepared dietary fiber is sealed and stored at low temperature. The basic nutritional components of BSDF freeze-dried powder were determined before the subsequent experiments (Supplementary Table S1).

### 2.2. Animals and diets

Animal experiments (1): 5-week-old male C57BL/6J mice (Vital River Laboratory Animal Technology Co., Beijing, China) were purchased and raised in a fixed environment (12 h light/dark cycle, 25 ± 2°C, 55% ± 10% humidity). After an adaptive feeding for 1 week, the mice were randomly divided into three groups: normal control diet (NCD), high-fat diet (HFD), high-fat diet supplemented with 6% BSDF freeze-dried powder (HFD-BSDF) (*n* = *8* per group) for 12 weeks. In this study, 6% BSDF was added based on the previous studies (14–16). Body weight and food intake were monitored weekly, and relevant tissues were collected at the end of the experiment. The company “Shuyishuer Biotech Co” provides all diets we need. At the same time, we show the specific compositions and energy densities of diets in Supplementary Table S2.

Animal experiments (2): Mices were administrated with high-fat diet (A-HFD) and high-fat diet supplemented with 6% BSDF freeze-dried powder (A-HFD-BSDF), respectively. Drinking water for both groups was replaced with sterile water containing a mixture of antibiotics. The antibiotic regulation determined from the previous literature (17), was composed of 125 g/mL ciprofloxine hydrocholoride, 100 μg/mL neomycin, 100 μg/mL metronidazole, 100 μg/mL cefazolin, 100 U/mL penicillin, 50 μg/mL streptomycin, 50 μg/mL vancomycin, and 1 mg/mL bacitracin, and replaced twice a week. Our animal experiments were supported by the Animal Protection Professional Committee of China Agricultural University (AW40601202-4-1).

### 2.3. Glucose tolerance test

The mouse intraperitoneal glucose tolerance test (ipGTT) was performed on the 11th week of the experiment. Mice fasted for 12 h and weighed, was intraperitoneally injected with glucose diluted solution (1.0 g/kg), and its glucose concentration was measured by glucose meter (Accu-chek, Roche, Switzerland).

The serum of mice was centrifuged (4°C, 10 min, 3000 rpm) after standing at 25 °C for 30 min, then determined with a mice insulin enzyme-linked immunosorbent assay (ELISA) kit (Alpco, USA). Homeostasis Model Assessment of Insulin Resistance (HOMA-IR) was calculated from fasting glucose and insulin (6).

### 2.4. Histological analysis

We stained liver tissue with both hematoxylin and eosin (H&E) and Oil Red O, but adipose tissue with H&E only, refers to the method of Ke et al. (18). Finally, the sections were observed and photographed under a high-level microscope.

### 2.5. Biochemical analysis

The concentrations of lipopolysaccharide (LPS) and tumor necrosis factor alpha (TNF-α) in serum were detected with commercial enzyme-linked immunosorbent assay (ELISA) kits (Enzyme Link Biotechnology Co., Ltd., Shanghai, China). Meanwhile, serum total triglycerides (TG), total cholesterol (TC), low density lipoprotein cholesterol (LDL-C), high density lipoprotein cholesterol (HDL-C), alanine aminotransferase (ALT) and aspartate aminotransferase (AST) was measured by the automatic biochemical analyzer (AU480, Olympus Corporation, Tokyo, Japan), refers to the method of Zhao et al. (1).

### 2.6. Gut microbiota analysis

We took 100 mg of mice fecal samples, extracted DNA, sequenced the amplification of the microbial 16S rRNA gene, and processed and analyzed the sequencing data to obtain the corresponding results. And details of the specific method are shown in the Supplementary method S1, refers to the method of previous researches (19–21).

### 2.7. RNA-Seq library preparation and sequencing

Total RNA was extracted from mouse liver tissue using TRIzol^®^ reagent according the manufacturer's instructions (Invitrogen). RNA quality was evaluated by electrophoresis using an Agilent 2100 Bioanalyzer (Agilent Technologies, San Diego, CA, USA). Samples with RNA integrity numbers (RINs) > 9.4 and with 260/280 nm absorbance ratios from 1.9 to 2.1 were used for the construction of RNA Seq libraries. Libraries were constructed using the TruSeqTM RNA Sample Prep kit (Illumina, San Diego, CA, USA) according to the manufacturer's instructions.

Sequencing of the libraries was performed on an Illumina HiSeq 2000 instrument by Shanghai Majorbio Biopharm Biotechnology (Shanghai, China), and individually assessed for quality using FastQC. Analysis of differential expression was carried out using DESeq2 (22). Statistical significance was assessed using a negative binomial Wald test, then corrected for multiple hypothesis testing with the Benjamini-Hochberg method. Functional enrichment cluster analysis was performed for Kyoto Encyclopedia of Genes and Genomes (KEGG) pathway enrichment analysis.

### 2.8. Real-time quantitative PCR

Target gene expressions were assessed using RT-qPCR on liver mRNA (6). The used primers are listed in Supplementary Table S3. The specific experimental steps are written in Supplementary method S2.

### 2.9. Quantification of fecal SCFAs

Determination of SCFAs in feces by gas chromatography-mass spectrometry (GC-MS) (23, 24). The specific experimental steps are written in Supplementary method S3.

### 2.10. Determination of metabolites in feces

The metabolites in faces were analyzed by LC-MS/MS (25). And details of the specific method shown in Supplementary method S4.

### 2.11. Statistical analysis

Statistical analyses were performed with the SPSS 25.0 software (SPSS Inc., Chicago, IL, USA). The data follow a normal distribution, and the variances of the groups are similar. Data are presented as Means ± SEM. Significant group differences were determined by one-way ANOVA followed by Duncan's test (*P* < 0.05). Differences between two groups were analyzed by an independent sample *t*-test (*P* < 0.05). Graphs were prepared using the Prism 7.0 software (La Jolla, CA, USA).

## 3. Results

### 3.1. Alleviation of HFD-induced obesity by BSDF supplementation

**Figure 1:**
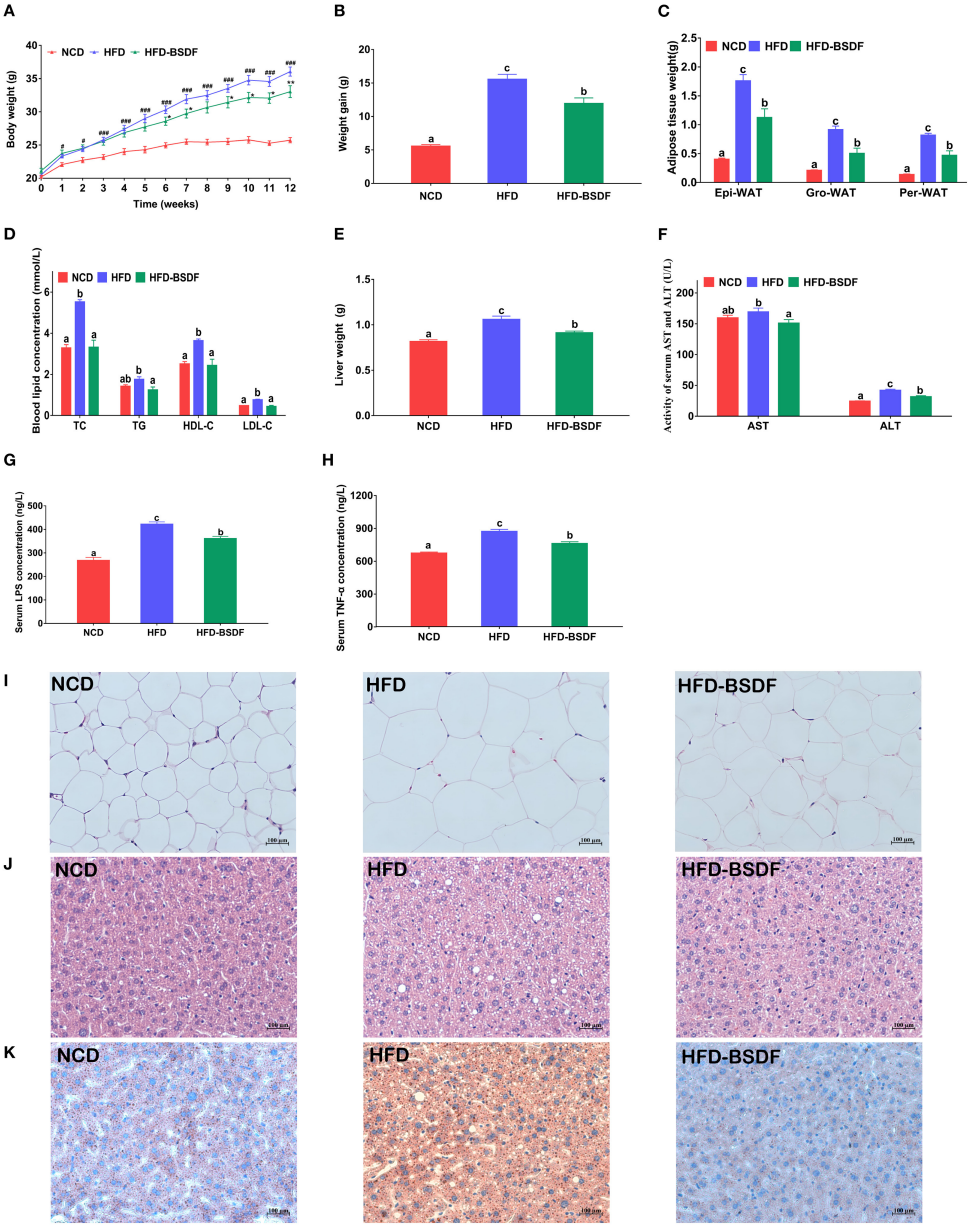
BSDF alleviation of HFD-induced obesity. **(A)** Body weight vs. time profiles; **(B)** weight gain; **(C)** white fat weight; **(D)** serum lipid level; **(E)** liver weight; **(F)** serum ALT and AST concentrations; **(G)** serum LPS concentration; **(H)** serum TNF-α concentration; **(I)** H&E staining of epididymal fat sections; **(J)** H&E staining of liver tissue; **(K)** Oil Red O staining of liver. Data presented as mean ± SEM, *n* = *8* per group. ^#^*p* < 0.05, ^###^*p* < 0.001, HFD vs. NCD; ^*^p < 0.05, ^**^p < 0.01, HFD–BSDF vs. HFD. a, b, c means in the same bar without a common letter differ at *p* < 0.05. Epi-WAT, epididymal fat; Per-WAT, perirenal fat; Gro-WAT, groin fat. NCD, normal control diet; HFD, high-fat diet; HFD-BSDF, high-fat diet supplemented with 6% BSDF freeze-dried powder.

Figure 1 showed the changes of related phenotypes after BSDF intervention in obese mice induced by HFD. It is noteworthy that at the beginning of the experiment, there was no significant difference in the initial body weight of NCD, HFD and HFD-BSDF mice. When the intervention time reached 6 weeks, the weight of mice in HFD-BSDF group was significantly lower than that in HFD group (*P* < 0.05) (Figure 1A). At the end of the experiment, the total weight gain of the HFD-BSDF was also significantly lower than that of the HFD group, which was consistent with the results of the graph of changes in mice body weight (*P* < 0.05) (Figure 1B). The weights of three types of white fat, namely epididymal fat, perirenal fat, and groin fat, were also analyzed and it was found that the HFD significantly increased the weight of all three types of white fat, with a significant decrease after the BSDF intervention (*P* < 0.05) (Figure 1C). The trends in energy efficiency in mice were consistent with the trends in body weight, weight gain, and white fat weight (*P* < 0.05) (Supplementary Figure S1A). However, there was no significant difference in energy intake between HFD-BSDF and HFD groups (*P* < 0.05) (Supplementary Figure S1B). This shows that the effect of BSDF on body weight and fat weight has nothing to do with food intake. In addition, BSDF significantly improved the elevated serum lipid levels TC, TG, HDL-C, LDL-C in high-fat mice (*P* < 0.05) (Figure 1D), and significantly reduced liver weight and liver function factors AST and ALT (*P* < 0.05) (Figures 1E, F). We then measured the inflammatory factors LPS and TNF-α in mice serum and found that BSDF was effective in improving overall inflammation levels in mice (*P* < 0.05) (Figures 1G, H). We further analyzed HE sections of epididymal fat and found that severe hypertrophy of white adipose tissue occurred in HFD mice and that BSDF intervention effectively ameliorated this tissue hypertrophy, which can be confirmed by the measurement of white fat area (Figure 1I, Supplementary Figure S1C). Following the observed reduction in liver weight, we made HE sections and oil-red sections of liver tissue, and the results showed that BSDF significantly improved liver steatosis and lipid droplet aggregation associated with a high-fat diet (Figures 1J, K). These results suggest that BSDF supplementation significantly ameliorated the abnormal weight gain, white fat accumulation and hepatic steatosis and tissue damage in HFD mice.

### 3.2. Improvement of insulin resistance and glucose tolerance insulin resistance in HFD mice by BSDF supplementation

**Figure 2:**
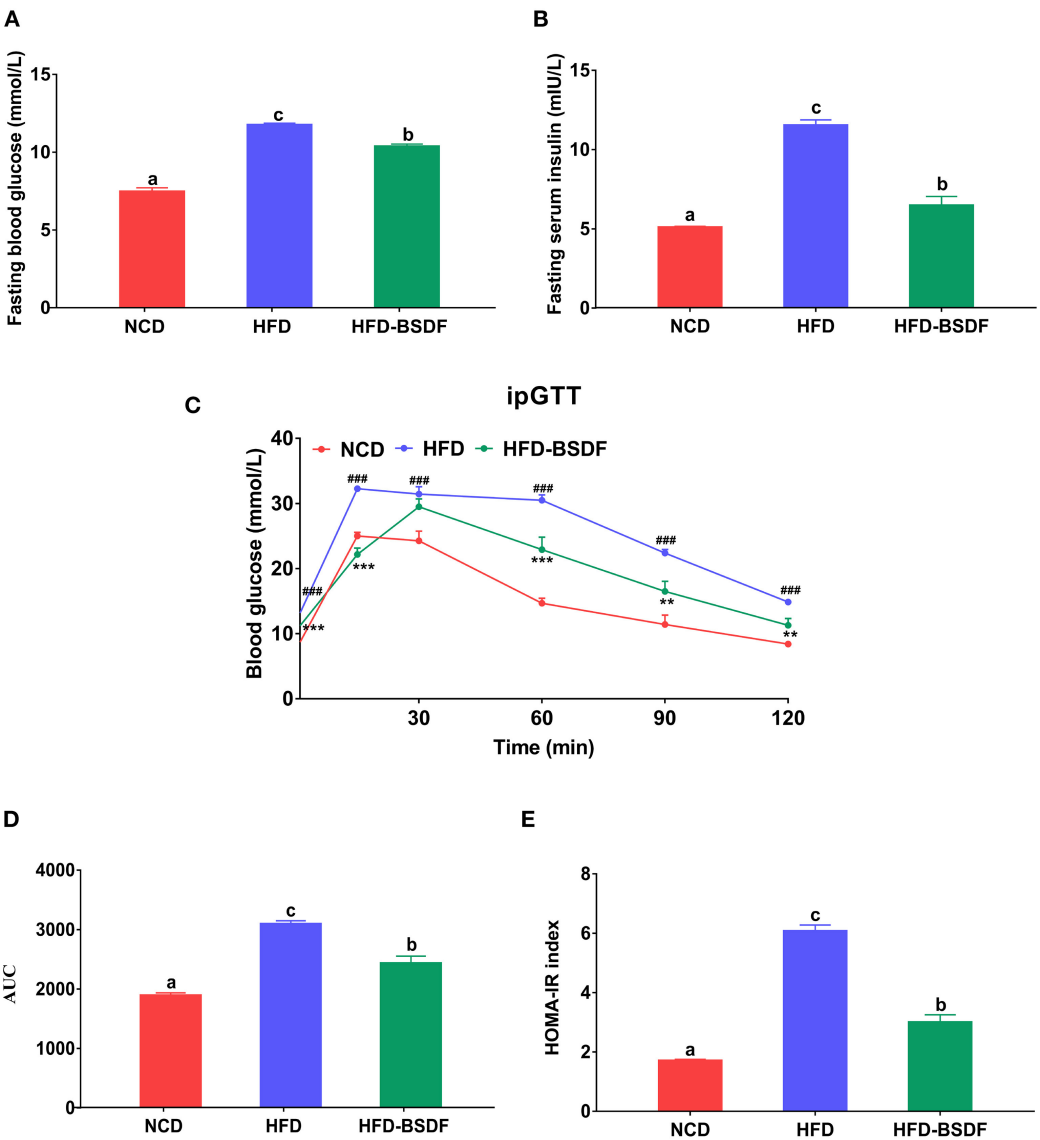
BSDF improved insulin resistance and glucose tolerance in HFD-fed mice. **(A)** Serum glucose, **(B)** serum insulin, **(C)** time courses of blood glucose levels in the intraperitoneal glucose tolerance test (iPGTT) **(D)** Area under the curve (AUC) of the blood glucose during ipGTT. **(E)** homeostatic model assessment for insulin resistance (HOMA-IR) index, and Data presented as mean ± SEM, *n* = *8*. ^###^*p* < 0.001, HFD vs. NCD; ^**^p < 0.01, ^***^p < 0.001, HFD-BSDF vs. HFD. a, b, c means in the same bar without a common letter differ at *p* < 0.05. NCD, normal control diet; HFD, high-fat diet; HFD-BSDF, high-fat diet supplemented with 6% BSDF freeze-dried powder.

The effect of BSDF on glucose metabolism in hyperlipidemic mice was shown in Figure 2. The fasting glucose of NCD mice was 7.59 ± 0.23 mmol/L, and that of HFD mice was 11.88 ± 0.10 mmol/L, which was significantly reduced to 10.49 ± 0.15 mmol/L after BSDF intervention (*P* < 0.05) (Figure 2A). Similarly, the fasting insulin of mice after BSDF intervention also decreased from 11.51 ± 0.34mIU/L to 6.46 ± 0.56mIU/L (*P* < 0.05) (Figure 2B), which indicates that the glucose and insulin regulation of obese mice is abnormal, and BSDF intervention can significantly alleviate the abnormal glucose metabolism. Glucose tolerance experiments also showed a similar effect, with the highest blood glucose values in the HFD group of mice at 15 min after glucose injection and a slow decline in blood glucose until 120 min later. However, after the BSDF intervention, the peak of blood glucose was delayed until 30 min, and the blood glucose values at 60 min, 90 min and 120 min were significantly lower than the corresponding blood glucose values in the HFD group, indicating that BSDF could effectively improve the glucose tolerance and enhance blood glucose regulation in high-fat mice (*P* < 0.05) (Figure 2C). Calculation of the area under the curve in Figure 2C further confirmed these results (*P* < 0.05) (Figure 2D), as did the trend in the HOMA-IR index (*P* < 0.05) (Figure 2E). These results show that BSDF is more effective in improving insulin resistance and regulating glucose homeostasis induced by high-fat diet.

### 3.3. Effects of BSDF on liver transcriptome in mice with HFD-induced obesity

**Figure 3:**
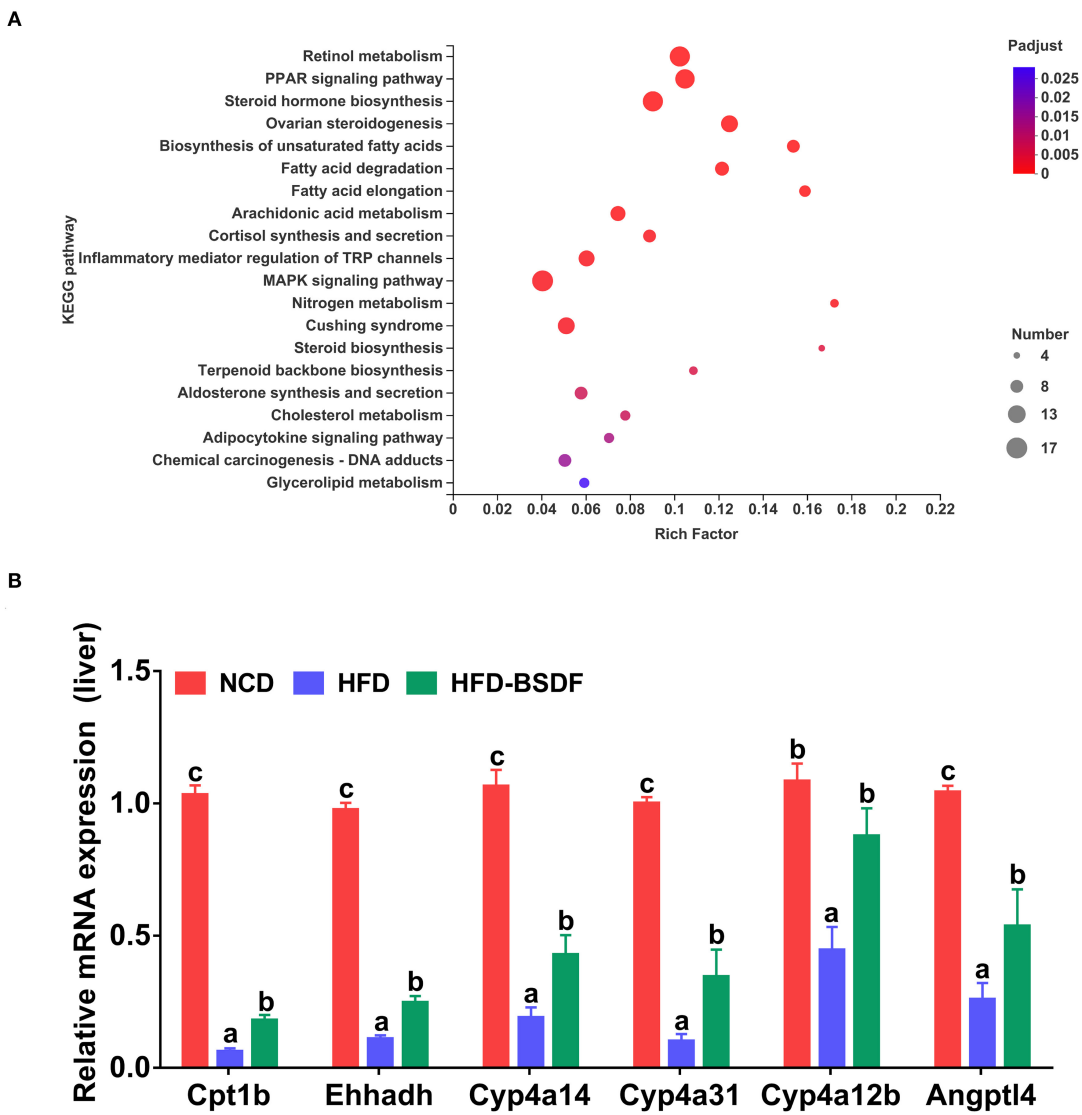
Effects of BSDF administration on the liver transcriptome in HFD-induced obese mice. **(A)** Kyoto Encyclopedia of Genes and Genomes (KEGG) pathway analysis using DEGs between t the NCD, HFD, and HFD-BSDF groups. *n* = 5 per group. **(B)** Relative mRNA expression of important genes on the PPAR signaling pathway in liver. *n* = *8* per group. Data presented as mean ± SEM, *n* = *8*. a, b, c means in the same bar without a common letter differ at *p* < 0.05. NCD, normal control diet; HFD, high-fat diet; HFD-BSDF, high-fat diet supplemented with 6% BSDF freeze-dried powder.

The liver transcriptome profiles in the NCD, HFD, and HFD-BSDF groups (*n* = 5) were compared using RNA-Seq to further assess how BSDF affected the whole-gene expression in HFD-induced obese mice. To recognize the differentially expressed genes (DEGs) among the three groups, we compared their transcriptomic profiles and analyzed the Kyoto Encyclopedia of Genes and Genomes (KEGG) pathways. The comparative analysis showed that BSDF was significantly involved in several metabolic pathways (*P*_adjust_ < 0.05), including retinol metabolism, peroxisome proliferator-activated receptor (PPAR) signaling pathway, steroid hormone biosynthesis, unsaturated fatty acid biosynthesis and fatty acid degradation (Figure 3A). Among these, the PPAR signaling pathway was the more significant signaling regulatory pathway. Combined with our research objectives and considering that the PPAR signaling pathway does play an important role in the regulation of lipid metabolism, we selected key genes on the PPAR signaling pathway for RT-qPCR experiments to validate and found that BSDF significantly increased the expression of Cpt1b, Ehhadh, Cyp4a14, Cyp4a31, Cyp4a31, Cyp4a12b, and other gene expression (*P* < 0.05) (Figure 3B). This is one of the mechanisms by which BSDF exerts its effect on improving obesity.

### 3.4. BSDF improvement of gut dysbiosis in obese mice

**Figure 4:**
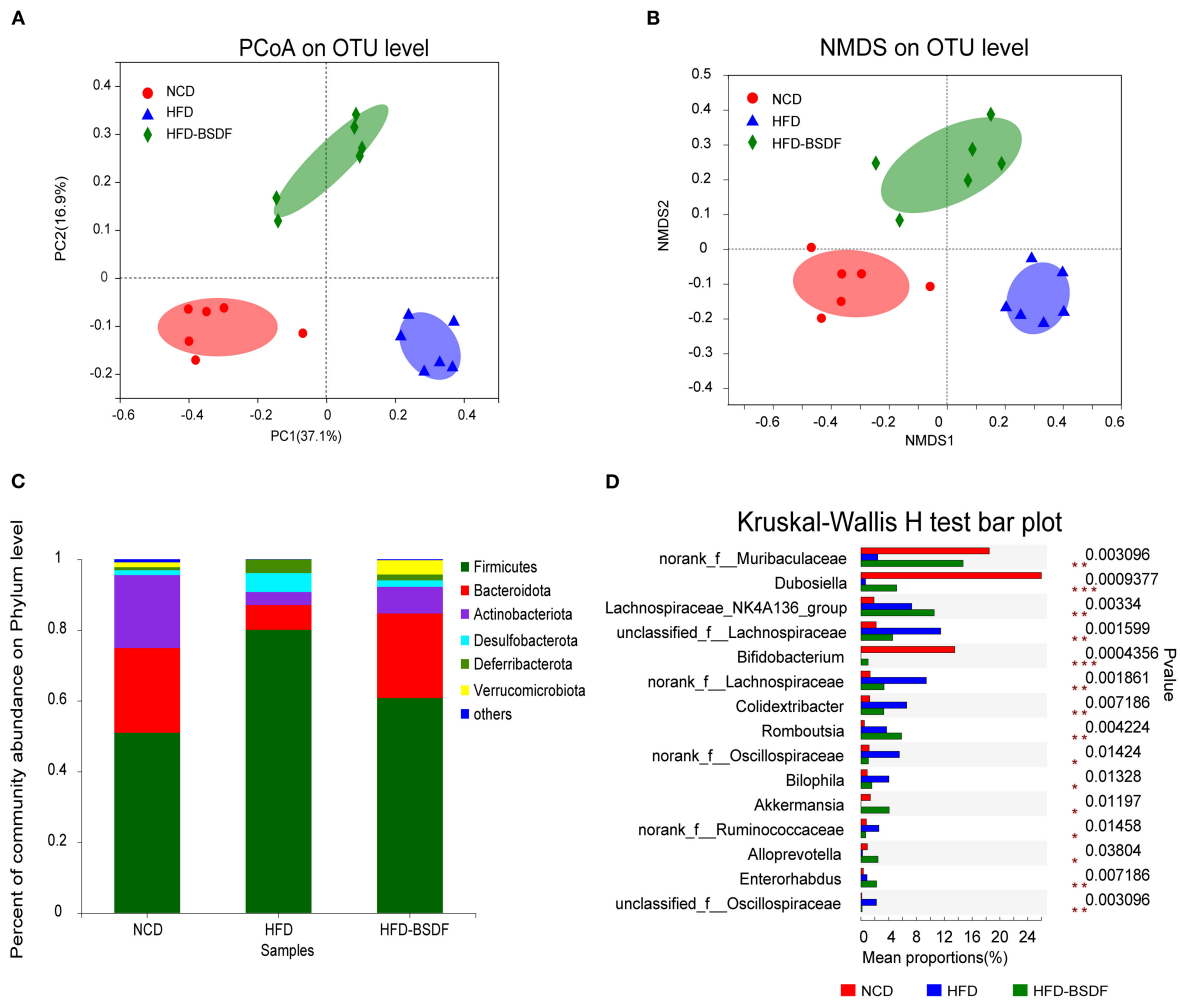
BSDF attenuated gut microbiota dysbiosis in HFD-fed mice. **(A, B)** PCoA and NMDS score plot based on Bray–Curtis. **(C, D)** The abundances of the gut microbiota at the phylum level, and the genus level. *n* = 6, per group. NCD, normal control diet; HFD, high-fat diet; HFD-BSDF, high-fat diet supplemented with 6% BSDF freeze-dried powder.

The gut microbiota disturbance caused by HFD was significantly improved after BSDF supplementation. The diversity index reflects the abundance and diversity of microbial communities, where Ace and Chao are indices of community richness and Shannon and Simpson are indices to evaluate community diversity. Compared to NCD mice, HFD mice showed significantly lower microbiota abundance and higher community diversity, but no significant difference from the HFD group after BSDF intervention (Supplementary Figures S2A–D). Bray-Curtis-based PCoA and NMDS could reflect the overall differences in OTU levels of microbes, and the results showed that the composition of gut microbiota was significantly changed after BSDF intervention (Figures 4A, B).

At the phylum level, HFD treatment increased the abundance of *Firmicutes* and *Desulfobacterota*, but caused the decrease in *Bacteroidota* and *Actinobacteriota* compared with the NCD. However, BSDF supplementation reversed this effect (Figure 4C). At the genus level, the HFD-BSDF diet led to a substantial increase in the *Bifidobacterium* and *Akkermansia* along with decreases in the *Bilophila, norankf_Ruminococcaceae* and *unclassified_f_Oscillospiraceae* abundances (Figure 4D). Herein, we used the LEfSe method to classify bacterial biomarkers from genus to phylum (Supplementary Figure S3A). The cladogram produced from the LEfSe analysis highlighted the dominant bacteria from the genus to the phylum level in each group. The LEfSe results (LDA > 3.5) indicated that the HFD led to greatly lower *Bifidobacterium* and *Dubosiella* levels and higher *Bilophila* and *Colidextribacter* abundances than the NCD group. Compared with the HFD group, BSDF supplementation not only reduces these effects, but also significantly increases *Akkermansia* and *Alloprevotella* abundances (Supplementary Figure S3B).

### 3.5. Effects of BSDF on fecal metabolomic profile in mice with HFD-induced obesity

**Figure 5:**
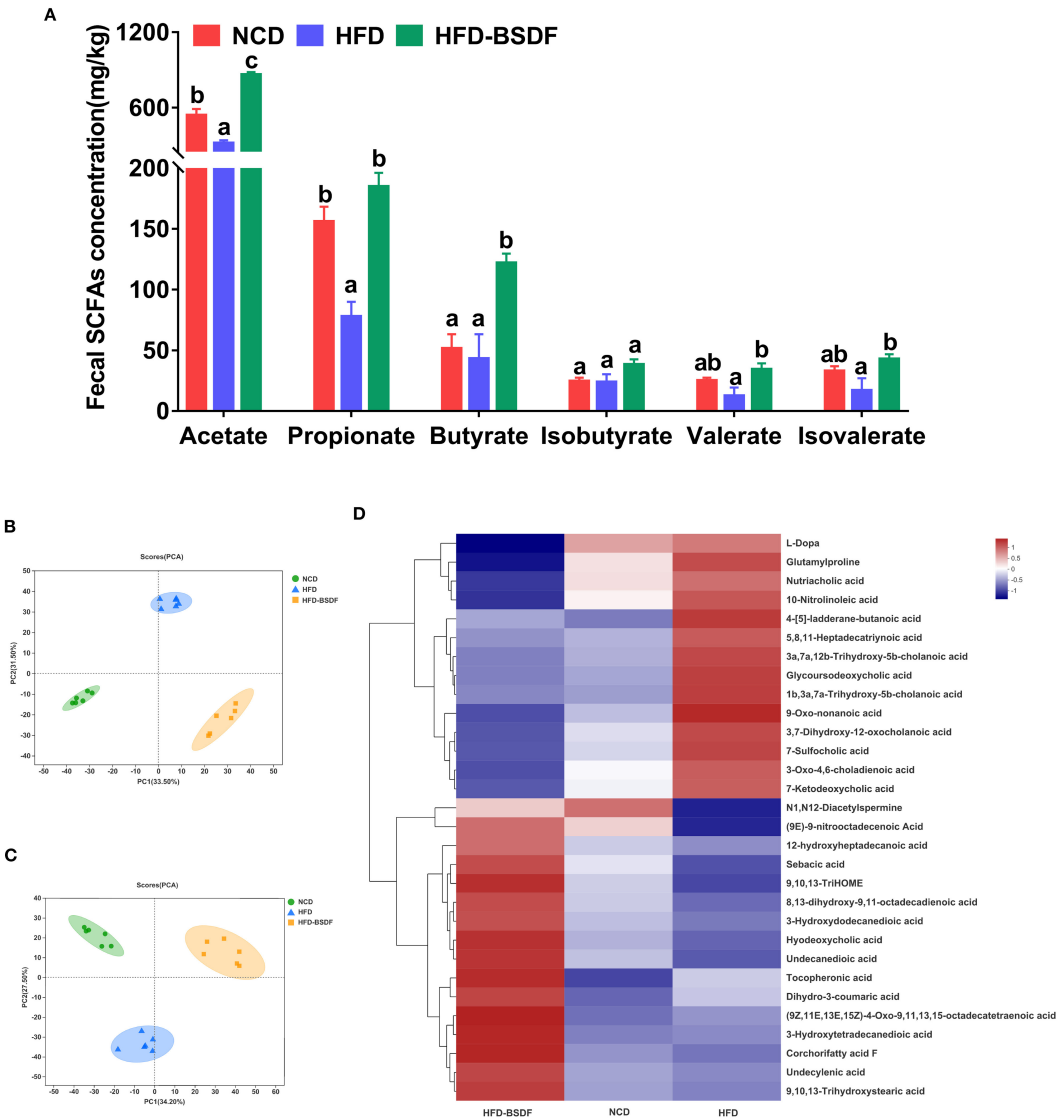
Effects of BSDF administration on fecal short chain fatty acids (SCFAs) and metabolomics in HFD-induced obese mice. **(A)** Concentration of SCFAs. *n* = *8* per group. Principal component analysis (PCA) score plots of fecal metabolomic profiles in all treatment groups. **(B)** Positive ion, and **(C)** negative ion. **(D)** Hierarchical cluster analyses of differential metabolites. Red and blue of increasing intensity indicate up-regulation or down-regulation, respectively. *n* = 6 per group. NCD, normal control diet; HFD, high-fat diet; HFD-BSDF, high-fat diet supplemented with 6% BSDF freeze-dried powder.

BSDF has an ameliorating effect on gut microbiota and exerts an ameliorating effect on obesity closely related to gut microbiota, then we further examined gut microbiota and host metabolites to facilitate a more comprehensive understanding of the mechanism of action of BSDF. We found that the concentrations of acetate and propionate in NCD mice were 516.25 ± 47.23 mg/kg and 146.26 ± 14.05 mg/kg, respectively. The concentrations of acetate and propionate in HFD mice were significantly reduced to 347.36 ± 34.89 mg/kg and 87.38 ± 13.88 mg/kg. After adding BSDF, the concentrations of acetate and propionate increased to 935.32 ± 27.35 mg/kg and 265.32 ± 12.54 mg/kg respectively, and the concentrations of butyrate, valerate and isovalerate were also significantly increased (*P* < 0.05) (Figure 5A). This suggests that BSDF supplementation can increase the production of SCFAs, which is beneficial to health.

Principal component analysis (PCA) of the electrospray ionization data in positive and negative ion mode showed the clear difference between the three diet groups (Figures 5B, C), indicating that BSDF has a significant effect on improving the overall metabolism of mice. Compared with the HFD group, BSDF supplementation resulted in significantly reduced abundances of 10-nitrolinoleic acid, l-dopa, 7-sulfocholic acid, 7-ketodeoxycholic acid, and nutriacholic acid, as well as significantly increased levels of tocopheronic acid, 9,10,13-trihydroxystearic acid, 9,10,13-trihydroxy-octadecenoic acid (9,10,13-TriHOME), corchorifatty acid F, undecanedioic acid, undecylenic acid, dihydro-3-coumaric acid, hyodeoxycholic acid, sebacic acid, and 3-hydroxydodecanedioic acid (Figure 5D). Above findings showed that BSDF supplementation can reverse the metabolic dysbiosis caused by HFD feeding.

### 3.6. Effects of BSDF on phenotype and insulin resistance of antibiotic-treated mice

**Figure 6:**
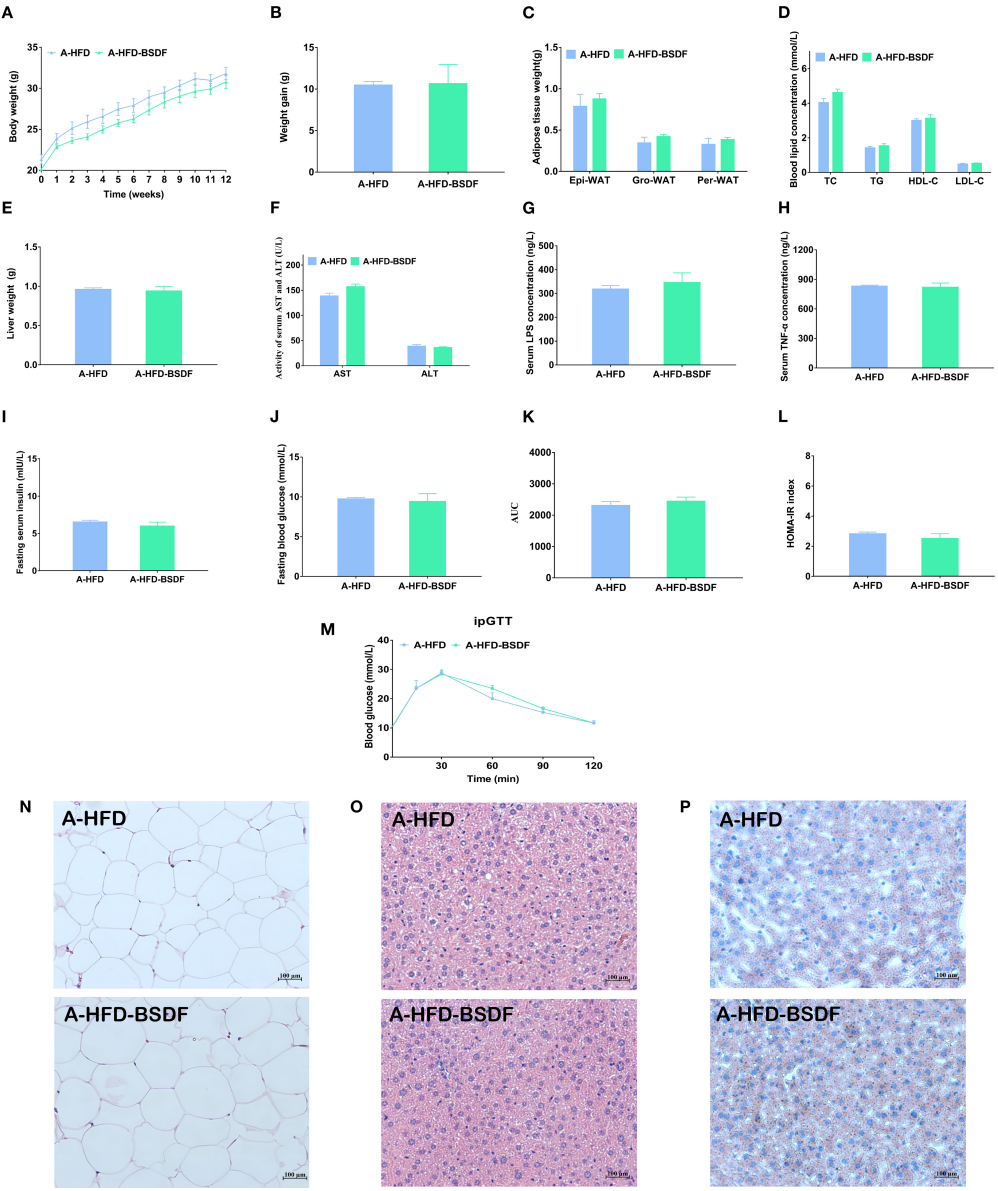
Effects of BSDF administration on phenotype and insulin resistance in antibiotic-treated mice. **(A)** Bodyweight time course measurements, **(B)** Weight gain, **(C)** White fat weight, **(D)** Serum lipid profile, **(E)** Liver weight, **(F)** Serum ALT and AST concentrations **(G)** Serum LPS concentrations. **(H)** Serum TNF-α concentrations. **(I)** Serum glucose, **(J)** Serum insulin, **(K)** Area under the curve (AUC) of the blood glucose during ipGTT. **(L)** Homeostatic model assessment for insulin resistance (HOMA-IR) index, **(M)** Time courses of blood glucose levels in the intraperitoneal glucose tolerance test (iPGTT), **(N)** H&E staining of epididymal fat sections. **(O)** H&E staining of liver tissue, and **(P)** Liver Oil Red O staining. Data presented as mean ± SEM, *n* = *8* per group. A-HFD vs. A-HFD-BSDF at *p* < 0.05. A-HFD, high-fat diet and antibiotic water; A-HFD-BSDF, high-fat diet supplemented with 6% BSDF freeze-dried powder and antibiotic water.

The sterile water containing broad-spectrum antibiotics was provided to A-HFD and A-HFD-BSDF groups for 12 weeks. The antibiotic water eliminated most of the gut microbiota, creating artificially germ-free mice. We found that there was never a significant difference in body weight between the A-HFD and A-HFD-BSDF groups of mice during the experiment (*P* < 0.05) (Figure 6A). At the end of the experiment, there was also no difference in the weight gain of the two groups (*P* < 0.05) (Figure 6B). There were also no differences in the three white fat weights, liver weight, lipid indices (TC,TG, HDL-C, LDL-C, AST and ALT), or inflammatory factors (*P* < 0.05) (Figures 6C–H). In addition, the differences in fasting insulin levels, fasting glucose, AUC, HOMA-IR index, changes in glucose tolerance and other glucose metabolism related indicators are all disappeared (*P* < 0.05) (Figures 6I–M). H&E staining of epididymal fat sections and liver and Oil Red O staining of liver further confirmed that there was no significant difference in the degree of white adiposity and hepatic steatosis (Figures 6N–P). This result was also supported by changes in white adipocyte area and feeding efficiency (Supplementary Figures S4A, B). These indirectly suggest that the beneficial effects of BSDF on weight loss are closely related to the gut microbiota.

## 4. Discussion

This study first assessed the effect of BSDF supplementation on improving obesity and related complications in HFD-fed mice. The results showed that BSDF significantly improved the phenotypic symptoms associated with obesity caused by weight gain, lipid accumulation, insulin resistance and inflammatory response (Figures 1, 2). In order to explain why BS can improve the obesity-related phenotype, we made a transcriptome analysis of mice liver. The results showed that the main metabolic pathway for BSDF to improve obesity is the PPAR signaling pathway, in which the differentially expressed genes were verified, which was consistent with the transcriptome analysis results (Figure 3). The effect of BSDF on gut microbiota was then investigated and BSDF was found to improve the gut micro-environment, promote the increase of beneficial microbiota such as Bifidobacterium and Akkermansia, and inhibit the growth of gut pathogenic microbiota (Figure 4). Next, we analyzed metabolites closely related to gut microbiota, and the results showed that BSDF also promoted the growth of SCFAs, enriched tocopheronic acid, 9,10,13-trihydroxystearic acid, 9,10,13-trihydroxy-octadecenoic acid (9,10,13-TriHOME), and other beneficial metabolites (Figure 5). Finally, to further investigate the role played by gut microbiota in the improvement of obesity by BSDF, this study allowed high-fat mice to drink broad-spectrum antibiotic water and found that there was no difference between BSDF intervention and A-HFD mice in artificially created germ-free mice, indicating the necessity of gut microbiota for the improvement of obesity by BSDF (Figure 6). Thus, it is proposed that the potential mechanism of BSDF to improve obesity is based on the composition of the gut microbiota and metabolic homeostasis.

### 4.1. BSDF and other dietary fiber have similar effects in alleviating obesity phenotype

Our results on the improvement of weight gain, lipid accumulation, elevated blood glucose and insulin resistance caused by obesity with different dietary fibers are consistent with the literature. A long-term study of high-fat induced obesity in C57BL/6J mice showed that the addition of oat insoluble fiber suppressed weight gain and lipid accumulation (26). Insoluble dietary fiber from enoki mushrooms, carrots and oats had hypoglycaemic and hypolipidaemic effects in both *in vitro* and *in vivo* experiments (27). Our results are consistent with all these studies. Interestingly, the dietary fiber we extracted from ciliated BS was also predominantly insoluble dietary fiber, and it has been reported in the literature that insoluble dietary fiber from Banner sweet dragon bamboo is more advantageous than other common dietary fibers in reducing body weight in obese mice (3), but the experimental period was only 6 weeks, and our experiment extended the experimental period to more comprehensively evaluate the beneficial effects of BSDF.

### 4.2. BSDF alleviates HFD-induced obesity by regulating PPAR signal pathway

In our study, BSDF significantly alleviated HFD-induced hepatic lipid accumulation and abnormal lipid metabolism by enhancing the PPAR/ fatty acid metabolic signaling pathway in the liver. The liver is a major regulatory organ of lipid metabolism, regulating various aspects of lipogenesis, fatty acid oxidation, lipoprotein uptake and secretion, and plays a key role in lipid metabolism (28–30). The transcriptome is also essential for interpreting the functional components of the genome, revealing the molecular composition of cells and tissues, and understanding development and disease (31–33), so we selected liver tissue for RNA-seq transcriptomic analysis to further investigate the potential molecular mechanisms underlying the anti-obesity effects of BSDF. The results revealed that peroxisome proliferator-activated receptor (PPAR) is the main signaling pathway involved in BSDF. Combined with our study objectives and considering that the PPAR signaling pathway does play an important role in the regulation of lipid metabolism, we selected key genes in the PPAR signaling pathway for validation by RT-qPCR experiments. Lipid metabolism in the liver is mainly regulated by the (peroxisome proliferator-activated receptor, PPAR) family (13, 34). When lipid accumulation occurs in the liver, it activates the PPAR signaling pathway, regulating the high expression of (Cytochrome P450 monoxygenase, CYP4A) enzymes, one of the most sensitive target genes of PPARα, and promoting fat energy expenditure (35). This may be the molecular mechanism by which BSDF acts.

### 4.3. BSDF has a broader effect on the gut microbiota than other insoluble dietary fibers

Analysis of changes in the gut microbiota and its metabolites (3), which are closely associated with the development of obesity, is important for understanding host metabolism and improving organismal health. Our study found that there is no significant difference between HFD group and HFD-BSDFgroup in diversity index. This suggests that the effect of BSDF on the gut microbiota of high-fat mice is not seen to differ in terms of abundance and diversity, and that there is something more worthy of analysis. At the phylum level, the abundance of *Bacteroidota* and *Actinobacteriota* was increased and the abundance of *Firmicutes* and *Desulfobacterota* was decreased in the HFD-BSDF group compared to the HFD group (Figure 4C). Several studies have shown that the *Bacteroidota* is less efficient at absorbing energy from food than *Firmicutes* (36), resulting in reduced calorie absorption and subsequent weight gain (37). The *Actinobacteria* (containing beneficial gut microbiota such as *Bifidobacterium*) has been reported to have a positive effect on host health (38). And the *Desulfobacterota* is associated with promoting LPS release, exacerbating inflammation, and leading to disturbed energy metabolism (39). These are consistent with our findings. At the genus level, populations of *Bifidobacterium* and *Akkermansia muciniphila* were increased after BSDF intervention (Figure 4D). Based on previous research, direct addition of *Bifidobacterium* and *Akkermansia* to the diet of C57BL/6J high-fat mice improved glucose tolerance and insulin sensitivity, reduced levels of the inflammatory factor TNF-α in obese mice (40, 41), and promoted acetate production (42). In addition, *Akkermansia muciniphila* also benefits propionate and butyrate production (43). These SCFAS are important gut metabolites that are beneficial for organismal health, such as reducing body mass in obese mice, improving disorders of glucose and lipid metabolism as well as fatty liver (44), anti-inflammatory (45). And in terms of the sources of dietary fiber, our literature research results found that soybean insoluble dietary fiber mainly increased the level of *Lachnospirace_Nk4A136_group* to improve obesity (8), and insoluble dietary fiber derived from brown seaweed Laminaria japonica only increased the level of *Akkermansia muciniphila* (9), while Banna sweet dragon bamboo insoluble dietary fiber reduced the level of *Akkermansia* levels and increased the levels of *Prevotella* (3). This shows that insoluble dietary fiber from different sources also differs in regulating gut microbiota, and even insoluble dietary fiber from different strains of bamboo shoots may have different results, suggesting that the structure of the BSDF may be intrinsic to their different functions.

### 4.4. BSDF promotes the production of metabolites related to the PPAR signaling pathway

Further measurements of SCFAS and other metabolites in feces revealed that BSDF improvement in obesity-promoting beneficial metabolite production was associated with activation of the PPAR signaling pathway. In this study, BSDF intervention significantly increased acetate, propionate, butyrate, isovalerate and valerate in mice feces (Figure 5A). This may be related to the fact that BSDF promoted an increase in beneficial bacteria (*Bifidobacterium* and *Akkermansia*) (42, 43). These SCFAs are the main products of dietary fiber fermentation and are thought to be associated with dietary fiber ameliorating metabolic diseases such as obesity. Notably, studies in the literature have indicated that SCFAs can induce a PPAR-dependent switch from lipid synthesis to utilization (46). Furthermore, fecal metabolomics results showed that BSDF intervention significantly increased a number of fatty acid (9-nitrooctadecenoic acid, 9,10,13-trihydroxystearic acid, 9,10,13-TriHOME, undecylenic acid, sebacic acid, and 3-hydroxydodecanedioic acid), bile acids (3a,7a,12b-trihydroxy-5b-cholanoic acid, 1b,3a,7a-trihydroxy-5b-cholanoic acid, and 7-sulfocholic acid) and other metabolites (Figure 5D). Some of these metabolites (Undecylenic acid, tocopheronic acid and corchorifatty acid F) have antioxidant activity that correlates with the fact that BSDF can improve inflammation (47–49). There are also some metabolites (sebacic acid and 3-hydroxydodecanedioic acid and 9-nitrooctadecenoic acid) associated with lowering blood glucose (50–52). However, it was brought to our attention that 9,10,13-trihydroxystearic acid (a stearic acid) and 9,10,13-TriHOME (a metabolite of linoleic acid) were effective in lowering cholesterol, an effect associated with modulation of the PPAR signaling pathway. 9,10,13-TriHOME is a downstream metabolite of linoleic acid, which is a natural ligand for PPAR and can directly activate the PPAR signaling pathway (53).

### 4.5. Antibiotic experiments proved the necessity of gut microbiota

Having observed the extensive effects of BSDF on the gut microbiota and metabolites of mice, we further used antibiotic experiments to investigate the important role of the microbiota. The antibiotic experiment is a well-established technique to study the effects of different diets on artificially germ-free mice by creating artificially germ-free mice after they have been orally administered a mixture of broad-spectrum antibiotics to clear most of the flora in the gut (17, 54). To determine whether the effect of BSDF in improving obesity in high-fat mice is dependent on the gut microbiota, we gave a mixture of broad-spectrum antibiotics orally to mice in the HFD and HFD-BSDF groups and ended the experiment after 12 weeks of feeding. After antibiotic treatment, A-HFD and A-HFD- BSDF mice showed a significant decrease in gut microbiota abundance and no significant differences in body weight, white fat weight, liver and adipose tissue lipid accumulation, or glucose homeostasis. These findings are consistent with recent studies. Oral antibiotics reduced body weight and adiposity index in HFD-fed mice, along with an increase in white fat lipolysis genes and a decrease in liver lipogenesis genes (55). Kevin et al. (54) also demonstrated that gut microbiota is an independent factor affecting insulin clearance in obese mice (54). After gavage of sterile filtered donor fecal suspension (FVT) from a lean donor in a diet-induced obese mouse model by Rasmussen et al. (56) obese mice showed reduced weight gain and normalized plasma glucose tolerance, but the beneficial effects associated with FVT were counteracted if the recipient mice were treated with antibiotics prior to FVT (56). The above suggests that the obesity ameliorating effect of BSDF disappears after removal of gut microbiota, laterally indicating the need for gut microbiota.

In conclusion, our research shows that BSDF supplementation can improve obesity induced by high-fat diet and its accompanying metabolic changes. This effect is related to regulating gut microbiota and PPAR/fatty acid metabolism signal pathway. Considering the limitations of antibiotic experiments, fecal microbiota transplantation (FMT) experiments are needed to verify these experimental results. The mechanism of BSDF improving lipid metabolism by regulating gut microbiota can be further studied. However, according to our existing research results, we suggest that the development and utilization of dietary resources of BS can be increased to give full play to the potential prebiotic value of BSDF to improve obesity and metabolic diseases.

## Data availability statement

The original contributions presented in the study are included in the article/Supplementary material, further inquiries can be directed to the corresponding author.

## Ethics statement

The animal study was reviewed and approved by the Animal Protection Professional Committee of China Agricultural University.

## Author contributions

XZ: conceptualization, methodology, investigation, data curation, and writing-original draft. LM, LD, DL, and FC: supervision. XH: writing—review and editing and funding acquisition. All authors contributed to the article and approved the submitted version.
