# Supplementary material for: Bamboo shoot dietary fiber alleviates gut microbiota dysbiosis and modulates liver fatty acid metabolism in mice with high-fat diet-induced obesity
Source: Front Nutr. 2023 Mar 9;10:1161698. doi: 10.3389/fnut.2023.1161698 (PMC10035599; doi:10.3389/fnut.2023.1161698)
Supplement: Supplementary file 1 [file Data_Sheet_1.docx]

Supplementary Material

Bamboo shoot dietary fiber alleviates gut microbiota dysbiosis and modulates liver fatty acid metabolism in mice with high-fat diet-induced obesity

Xiaolu Zhou, Lingjun Ma, Li Dong, Daotong Li, Fang Chen, Xiaosong Hu*

*** Correspondence:** Prof. Xiaosong Hu: huxiaos@263.net (X. Hu).

# Supplementary Method

## Method S1. Gut Microbiota Analysis.

Total microbial genomic DNA was extracted from fecal samples using the E.Z.N.A.^®^ soil DNA Kit (Omega Bio-tek, Norcross, GA, U.S.) according to manufacturer’s instruc-tions. The quality and concentration of DNA were determined by 1.0% agarose gel elec-trophoresis and a NanoDrop^®^ ND-2000 spectrophotometer (Thermo Scientific Inc., USA) and kept at -80 °C prior to further use. The hypervariable region V3-V4 of the bacterial 16S rRNA gene were amplified with primer pairs 338F (5'-ACTCCTACGGGAGGCAGCAG-3') and 806R (5'-GGACTACHVGGGTWTCTAAT-3') by an ABI GeneAmp® 9700 PCR ther-mocycler (ABI, CA, USA). The PCR reaction mixture including 4 μL 5 × Fast Pfu buffer, 2 μL 2.5 mM dNTPs, 0.8 μL each primer (5 μM), 0.4 μL Fast Pfu polymerase, 10 ng of tem-plate DNA, and ddH2O to a final volume of 20 µL. PCR amplification cycling conditions were as follows: initial denaturation at 95 °C for 3 min, followed by 27 cycles of denatur-ing at 95 °C for 30 s, annealing at 55 °C for 30 s and extension at 72 °C for 45 s, and sin-gle extension at 72 °C for 10 min, and end at 4 °C. All samples were amplified in tripli-cate. The PCR product was extracted from 2% agarose gel and purified using the AxyPrep DNA Gel Extraction Kit (Axygen Biosciences, Union City, CA, USA) according to manu-facturer’s instructions and quantified using Quantus™ Fluorometer (Promega, USA).

Purified amplicons were pooled in equimolar amounts and paired-end sequenced on an Illumina MiSeq PE300 platform/NovaSeq PE250 platform (Illumina, San Diego,USA) according to the standard protocols by Majorbio Bio-Pharm Technology Co. Ltd. (Shang-hai, China).

Raw FASTQ files were de-multiplexed using an in-house perl script, and then quali-ty-filtered by fastp version 0.19.6 and merged by FLASH version 1.2.7 with the follow-ing criteria: (i) the 300 bp reads were truncated at any site receiving an average quality score of < 20 over a 50 bp sliding window, and the truncated reads shorter than 50 bp were discarded, reads containing ambiguous characters were also discarded; (ii) only overlap-ping sequences longer than 10 bp were assembled according to their overlapped sequence. The maximum mismatch ratio of overlap region is 0.2. Reads that could not be assembled were discarded; (iii) Samples were distinguished according to the barcode and primers, and the sequence direction was adjusted, exact barcode matching, 2 nucleotide mis-matches in primer matching. Then the optimized sequences were clustered into opera-tional taxonomic units (OTUs) using UPARSE 7.1 with 97% sequence similarity level. The most abundant sequence for each OTU was selected as a representative sequence. The OTU table was manually filtered, i.e., chloroplast sequences in all samples were removed. To minimize the effects of sequencing depth on alpha and beta diversity measure, the number of 16S rRNA gene sequences from each sample were rarefied to 20,000, which still yielded an average Good’s coverage of 99.09%, respectively. The taxonomy of each OTU representative sequence was analyzed by RDP Classifier version 2.2 against the 16S rRNA gene database (eg. Silva v138) using confidence threshold of 0.7.

Bioinformatic analysis of the gut microbiota was carried out using the Majorbio Cloud platform (https://cloud.majorbio.com). The alpha diversity analysis was performed us-ing Mothur (version V. 1. 30. 2). Both nonmetric multidimensional scaling (NMDS) and principal coordinate analysis (PCoA) were applied to quantify the compositional differ-ences between the microbial communities. They based on Bray-curtis dissimilarity using Vegan v2.5-3 package. ANOSIM analysis was used to test for significant differences in clusters among the groups. The relative abundances of different bacterial communities were assessed with the Wilcoxon rank-sum test at a confident level of 95%, which is corrected by false discovery rate (FDR). The linear dis-criminant analysis (LDA) effect size (LEfSe) (http://huttenhower.sph.harvard.edu/LEfSe) was performed to identify the significantly abundant taxa (phylum to genus) of bacteria among the different groups (LDA score > 3, p < 0.05, FDR corrected). LEfSe were assessed with the non-parametric factorial Kruskal-Wallis (KW) sum-rank test.

## Method S2. Real-Time Quantitative PCR

Target gene expressions were assessed using RT-qPCR on liver mRNA, which was isolated by a Trizol reagent (Invitrogen, Waltham, MA, USA), purified by a RNeasy Mini Kit (Qiagen, Venlo, Netherlands) and reverse transcribed with a FastQuant RT Kit (TianGen, Beijing, China). Template cDNA (1.5 μL) was mixed with 0.5 μL primers, 5 μL SYBR Green I Master Mix (Roche Diagnostics, Basel, Switzerland) and 3 uL water. PCRs were performed in triplicates on a LightCycler 480 Real-Time PCR system following this program: initial denaturation at 95 °C for 10 min, 35 PCR cycles of 95 °C for 10 s, 60 °C for 30 s, 72 °C for 15 s, followed by a melting curve. The used primers are listed in Supplementary Table S3. Relative quantification was calculated by the comparative 2−ΔΔCt method with 6 biological replicates and was normalized against GAPDH gene expression. Mean expression level of NCD mice was set at a value of 1 for data normalization.

## Method S3. Quantification of fecal Short-chain fatty acids (SCFAs).

1. Sample Preparation of Feces

Prepare an aqueous extract of feces. Weigh feces (approximately 50 mg mouse feces) in a 1.5 mL plastic tube with 0.1 mg accuracy and add 300 μL water. Homogenize the sample using a bullet blender: add two clean 3.2 mm steel beads and blend the sample for 5 min. Centrifuge at 1400 × g for 10 min. Transfer the supernatant to a fresh 1.5 mL plastic tube.

Prepare a glass autosampler vial for every sample. For calibration samples, add 250 uL acetone, 10 μL 1 ug/mL IS solution, and 10 uL of the calibration series SCFA standards at the desired concentration. In case of feces analysis, add 10 uL water which is preprocessed exactly the same as the biological samples. For biological samples, add 250 uL acetone, 10 μL 1 ug/mL IS solution inEtOH, 10 μL EtOH, and 10 uL aqueous feces into a glass autosampler vial. For blank samples, add 250 uL acetone, 10 μL 1 ug/mL IS solution, and 10 uL EtOH into a glass autosampler vial. In case of feces analysis, 10 uL water should be added which is preprocessed in exact the same way as the biological samples. For every type of biological matrix used in an experiment, three blank samples should be included. Vortex all samples. Add 100 μL 172 mM PFBBr in acetone. Vortex all samples. Heat the samples at 60 °C for 30 min in a laboratory stove. Let the samples cool down to room temperature (approximately 15 min). Add 500 uL n-hexane and 250 uL water to the samples. Shake the vial in vertical direction for approximately 10 s. Let the samples rest for 1 min at room temperature. Prepare a new empty glass autosampler vial with a glass insert for every sample. Transfer 250 μL of the n-hexane (upper layer) into the glass insert.

1. GC-MS Analysis

Inject 1 uL in the GC-MS, splitless at 280 °C.Use helium as carrier gas at a constant flow rate of 1.20 mL/min.Use the following temperature gradient: 1 min at 40 °C, linear increase at 40 °C/min to 60 °C, held for 3 min at 60 °C, linear increase at 25 °C/min to 210 °C, linear increase at 40 °C/min to 315 °C, and held for 3 min at 315 °C.Set the transfer line temperature at 280 °C. Keep the ionization source temperature at 280 °C.Use methane as chemical ionization gas at approximately 15 psi. Detect ions obtained in the negative mode using SIM. As a consequence of small chromatographic differences (e.g., GC column length), the exact RT varies between various GC systems. Hence, calibration using external standards is mandatory.

1. Data Analysis

Integrate the obtained signal.Calculate the relative retention time (RRT) and area ratios using the respective IS. Determine the slope and LLOQ for every SCFA by performing linear regression. It is recommended to use a weighing factor of 1/x2. Calculate the SCFA concentrations by using the area ratios obtained from the biological samples, average signal of the blank samples as intercept, and the slopes obtained from the analysis of the calibration series samples. Take into account the sample dilution for feces.

## Method S4. Determination of metabolites in faces.

50 mg solid sample were accurately weighed, and the metabolites were extracted using a 400 µL methanol: water (4:1, v/v) solution. The mixture was allowed to settle at -20 °C and treated by High-throughput tissue crusher Wonbio-96c (Shanghai Wanbo biotechnology co., LTD) at 50 Hz for 6 min, followed by vortexing for 30 s and ultrasound at 40 kHz for 30 min at 5 °C. The samples were placed at -20 °C for 30 min to precipitate the proteins. After centrifugation at 13,000 g at 4 °C for 15 min, the supernatant was carefully transferred to sample vials for LC-MS/MS analysis.

Chromatographic separation of the metabolites was performed on a Thermo UHPLC system equipped with an ACQUITY BEH C18 column (100 mm × 2.1 mm i.d., 1.7 µm; Waters, Milford, USA). The mobile phases consisted of 0.1% formic acid in water (solvent A) and 0.1% formic acid in acetonitrile: isopropanol (1:1, v/v) (solvent B). The solvent gradient changed according to the following conditions: from 0 to 3 min, 95% (A): 5% (B) to 80% (A): 20% (B); from 3 to 9 min, 80% (A): 20% (B) to 5% (A): 95% (B); from 9 to 13 min, 5% (A): 95% (B) to 5% (A): 95% (B); from 13 to 13.1 min, 5% (A): 95% (B) to 95% (A): 5% (B); from 13.1 to 16 min, 95% (A): 5% (B) to 95% (A): 5% (B) for equilibrating the systems. The sample injection volume was 2 µL and the flow rate was set to 0.4 mL/min. The column temperature was maintained at 40°C. During the period of analysis, all these samples were stored at 4 °C.

The mass spectrometric data were collected using a Thermo UHPLC-Q Exactive Mass Spectrometer equipped with an electrospray ionization (ESI) source operating in either positive or negative ion mode. The optimal conditions were set as follows: Aus gas heater temperature, 400 °C; Sheath gas flow rate 40 psi; Aus gas flow rate 30 psi; ion-spray voltage floating (ISVF)-2800 V in negative mode and 3500 V in positive mode; Normalized collision energy, 20-40-60 V rolling for MS/MS. Data acquisition was performed with the Data Dependent Acquisition (DDA) mode. The detection was carried out over a mass range of 70-1050 m/z.

After UPLC-TOF/MS analyses, the raw data were imported into the Progenesis QI 2.3 (Nonlinear Dynamics, Waters, USA) for peak detection and alignment. The preprocessing results generated a data matrix that consisted of the retention time (RT), mass-to-charge ratio (m/z) values, and peak intensity. Metabolic features detected at least 80 % in any set of samples were retained. After filtering, minimum metabolite values were imputed for specific samples in which the metabolite levels fell below the lower limit of quantitation and each Metabolic features were normalized by sum. The internal standard was used for data QC (reproducibility), metabolic features which the relative standard deviation (RSD) of QC > 30% were discarded. Following normalization procedures and imputation, statistical analysis was performed on log transformed data to identify significant differences in metabolite levels between comparable groups. Mass spectra of these metabolic features were identified by using the accurate mass, MS/MS fragments spectra and isotope ratio difference with searching in reliable biochemical databases as Human metabolome database (HMDB) (http://www.hmdb.ca/) and Metlin database (https://metlin.scripps.edu/). Concretely, the mass tolerance between the measured m/z values and the exact mass of the components of interest was ± 10ppm. For metabolites having MS/MS confirmation, only the ones with MS/MS fragments score above 30 were considered as confidently identified. Otherwise, metabolites had only tentative assignments.

A multivariate statistical analysis was performed using ropls (Version1.6.2, http://bioconductor.org/packages/release/bioc/html/ropls.html) R package from Bioconductor on Majorbio Cloud Platform (https://cloud.majorbio.com). Partial least squares discriminate analysis (PLS-DA) was used for statistical analysis to determine global metabolic changes between comparable groups. All of the metabolite variables were scaled to pareto Scaling prior to conducting the PLS-DA. The model validity was evaluated from model parameters R2 and Q2, which provide information for the interpretability and predictability, respectively, of the model and aviod the risk of over-fitting. Variable importance in the projection (VIP) were calculated in PLS-DA model. p values were estimated with paired Student’s t-test on Single dimensional statistical analysis.

Statistically significant among groups were selected with VIP value more than 1 and p value less than 0.05 (FDR corrected). Differential metabolites among two groups were summarized, and mapped into their biochemical pathways through metabolic enrichment and pathway analysis based on database search (KEGG, http://www. genome.jp/kegg/). These metabolites can be classified according to the pathways they involved or the functions they performed. Enrichment analysis was usually to analyze a group of metabolites in a function node whether appears or not. The principle was that the annotation analysis of a single metabolite develops into an annotation analysis of a group of metabolites. scipy.stats (Python packages) ( https://docs.scipy.org/doc/scipy/ ) was exploited to identify statistically significantly enriched pathway using Fisher’s exact test.

# Supplementary Figures


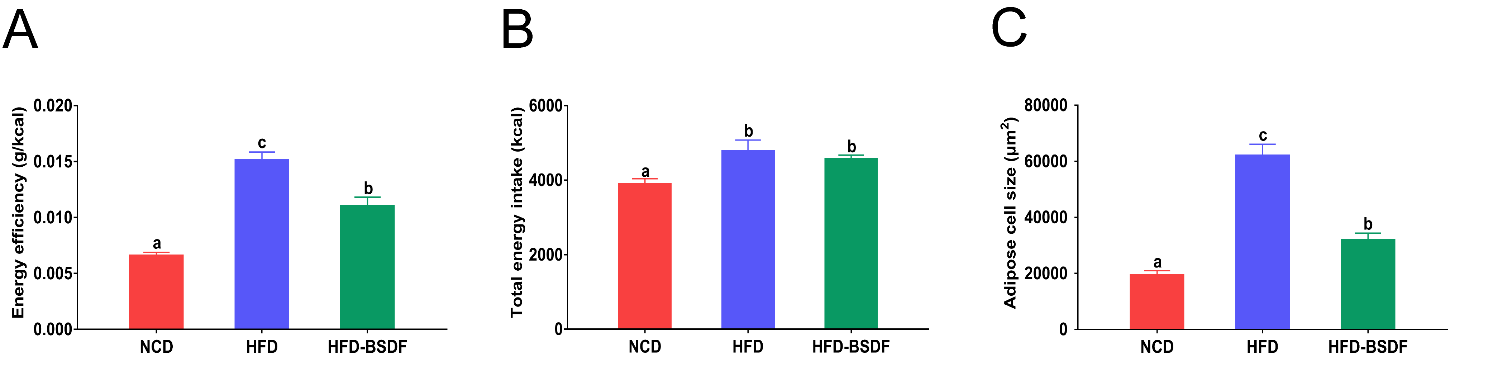


**Supplementary Figure 1.** Effects of BSDF on (A) Energy efficiency (B) Energy intake, and (C) Adipose cell size. Values expressed as mean ± SEM, n = 8 per group. a, b, c means in the same bar without a common letter differ at *p* < 0. 05.


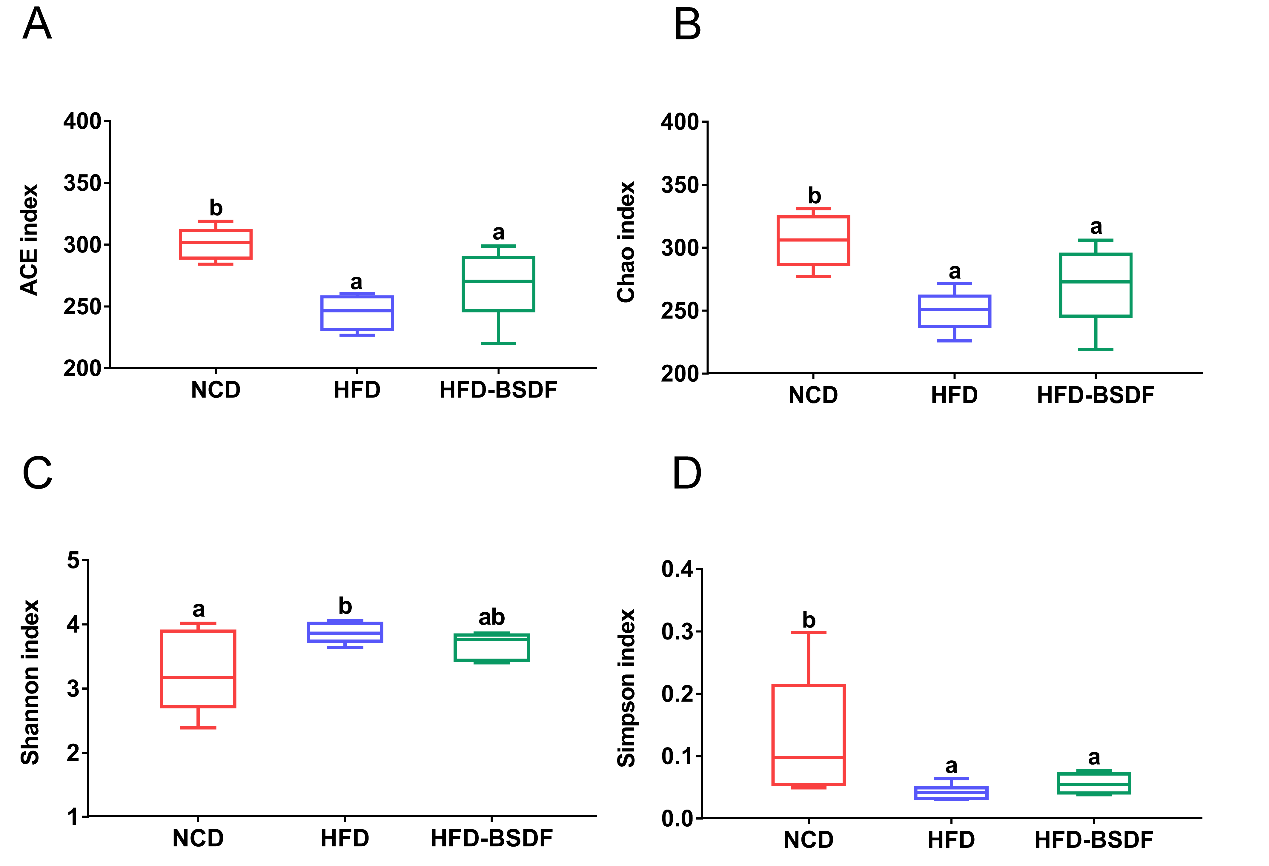


**Supplementary Figure 2.** BSDF induced gut microbial changes in mice. (A, B) The community richness accessed by the ACE and Chao indices. (C, D) The community diversity accessed by the Shannon and Simpson indices. Values expressed as mean ± SEM, n = 6 per group. a, b, c means in the same bar without a common letter differ at *p* < 0. 05.

**A**


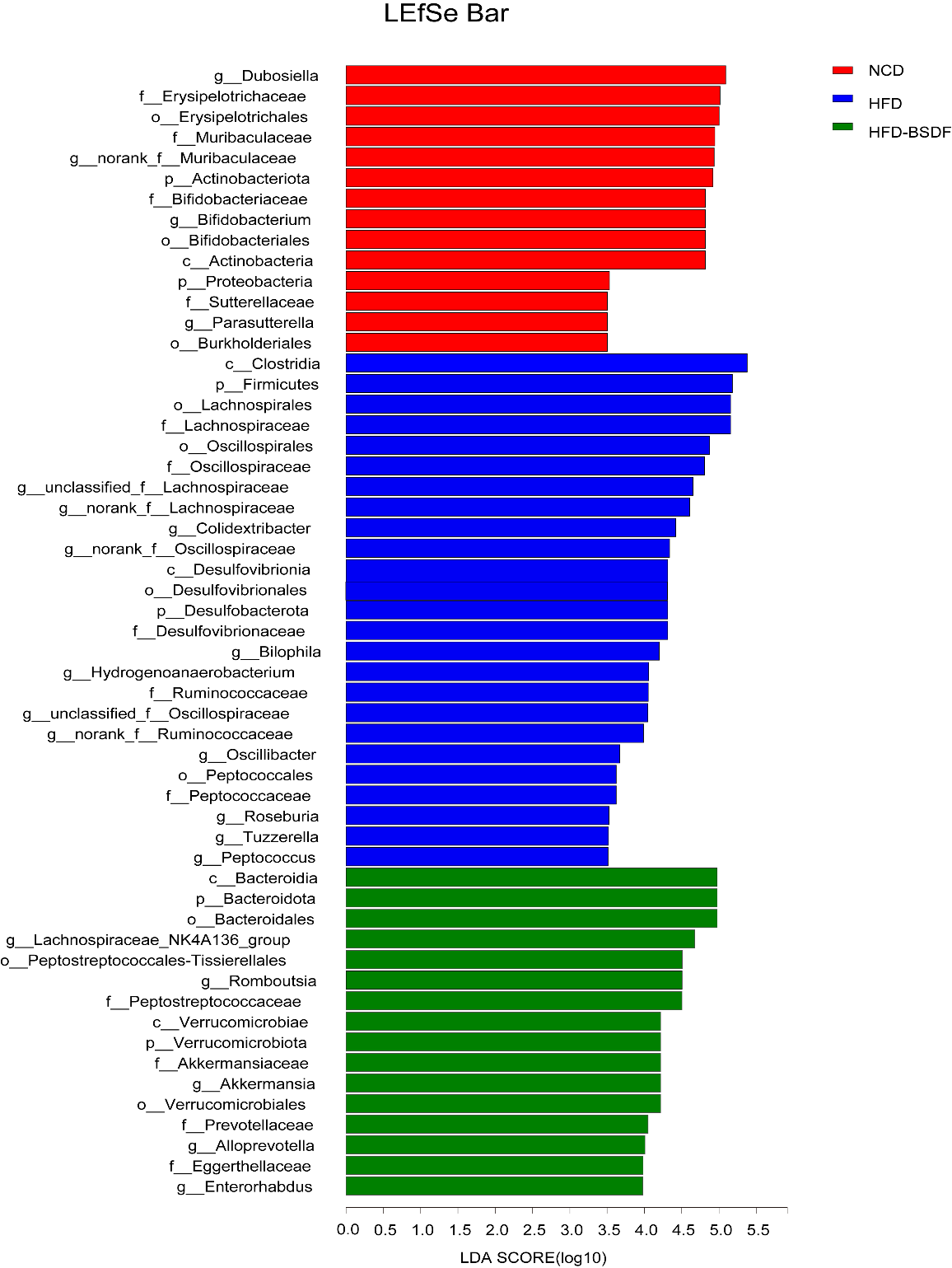


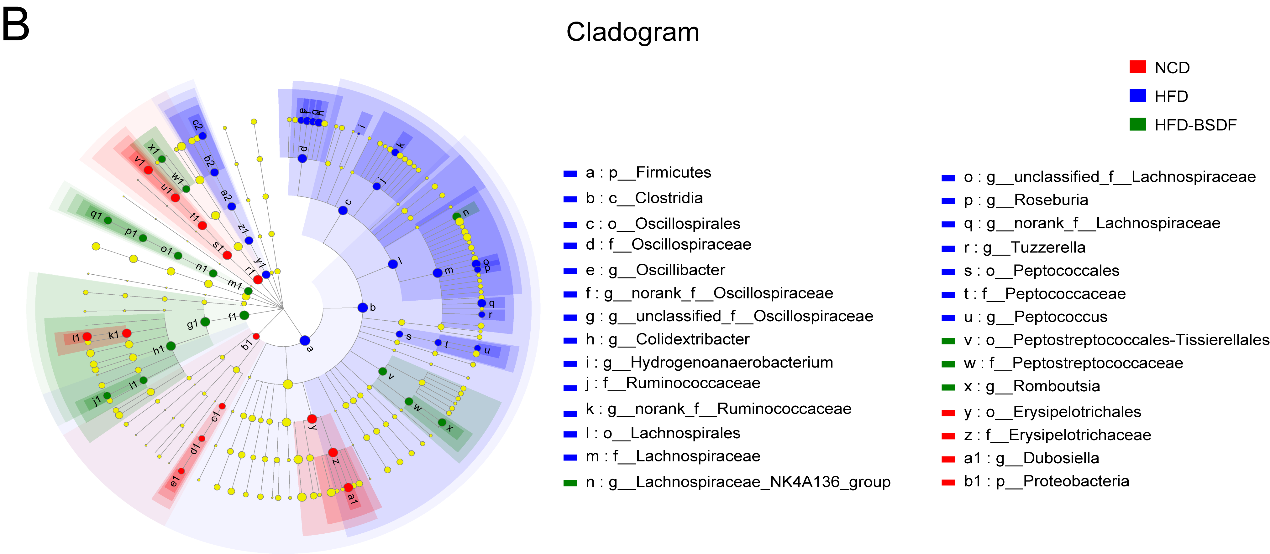


**Supplementary Figure 3.** (A) Linear discriminant analysis (LDA) scores derived from LEfSe analysis, showing the biomarker taxa LDA score of >3.5. (B) Cladogram plot from LEfSe analysis. The dots with different colors represented the important bacteria in each group with the same color. The yellow dots represented the unimportant bacteria that could not affect the gut microbiota community within groups. n = 6 per group.


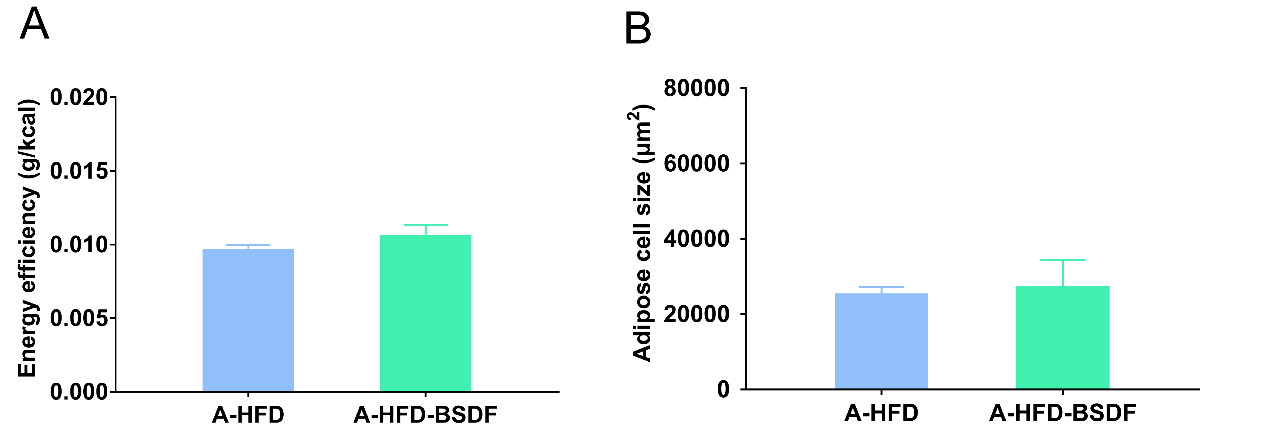


**Supplementary Figure 4.** Effects of BSDF on (A) Energy intake, and (B) Adipose cell size, in antibiotic-treated mice. Values expressed as mean ± SEM, n = 8 per group. a, b, c means in the same bar without a common letter differ at *p* < 0. 05.

# Supplementary Tables

**Supplementary Table 1.** Composition of Bamboo shoots Dietary fiber freeze-dried powder (g/100g)

| nutrient content | Dry weight |
| --- | --- |
| Total dietary fiber | 72.50±3. 40 |
| Insoluble dietary fiber | 0.40±0. 07 |
| Soluble dietary fiber | 72.1±1. 56 |
| protein | 7.83±0. 99 |
| fat | 6.5±0. 03 |
| carbohydrate | 0.1±0. 03 |
| moisture | 4.61±0. 06 |
| ash | 8.5±0. 40 |

Data are expressed as mean ± standard deviation (n = 3).

**Supplementary Table 2.** Composition of experimental diets^a^

| Ingredient (g/kg) | NCD^b^ | HFD | HFD-BSDF |
| --- | --- | --- | --- |
| Bamboo shoots Dietary fiber freeze-dried powder | 0 | 0 | 60.21 |
| Casein, 80 Mesh | 189. 56 | 258. 45 | 253.02 |
| L-Cystine | 2. 84 | 3. 88 | 3. 88 |
| Corn Starch | 479. 79 | 0. 00 | 0. 00 |
| Maltodextrin 10 | 118.48 | 161. 53 | 161.47 |
| Sucrose | 73. 74 | 88. 91 | 88. 91 |
| Cellulose | 47. 39 | 64. 61 | 14.40 |
| Soybean Oil | 23. 70 | 32. 31 | 27. 81 |
| Lard | 18. 96 | 316. 60 | 316. 60 |
| Mineral Mix, S10026 | 9. 48 | 12. 92 | 12. 92 |
| DiCalcium Phosphate | 12. 32 | 16. 80 | 16. 80 |
| Calcium Carbonate | 5. 21 | 7. 11 | 7. 11 |
| Potassium Citrate, 1 H20 | 15. 64 | 21. 32 | 21. 32 |
| Vitamin Mix, V10001C | 0. 95 | 12. 92 | 12. 92 |
| Choline Bitartrate | 1. 90 | 2.58 | 2.58 |
| FD&C Blue Dye | 0. 01 | 0. 00 | 0. 00 |
| FD&C Yellow Dye | 0. 04 | 0. 00 | 0. 00 |
| FD&C Blue Dye #1 | 0. 00 | 0. 005 | 0. 005 |
| % Energy and source | | | |
| Protein | 20 | 20 | 20 |
| Carbohydrate | 70 | 20 | 20 |
| Fat | 10 | 60 | 60 |

^a^ Diet formulae of NCD and HFD and the ingredients were obtained from the Shuyishuer Biotech Co., Ltd,Changzhou, China.

^b^ NCD, normal control diet; HFD, high-fat diet; HFD-BSDF, high-fat diet supplemented with freeze-dried powder of bamboo shoots dietary fiber.

**Supplementary Table 3.** Primer sequences

| Gene | Forward sequence (5'-3') | Reverse sequence (5'-3') |
| --- | --- | --- |
| GAPDH | AGGTCGGTGTGAACGGATTTG | GGGGTCGTTGATGGCAACA |
| Cpt1b | TCTTCTTCCGACAAACCCTGA | GAGACGGACACAGATAGCCC |
| Ehhadh | ATGGCTGAGTATCTGAGGCTG | ACCGTATGGTCCAAACTAGCTT |
| Cyp4a14 | TCTGGGTTCTTCCAATGGGC | GGACTCGTATATTGCTCCCCG |
| Cyp4a31 | CATCACCGCCCTTTCACTG | TCCCCCAGAACCATCGAGG |
| Cyp4a12b | GGGGAGATCAGACCCAAAAGC | ATTCGTCGGTGCTGAAACCAT |
| Angptl4 | GCATCCTGGGACGAGATGAAC | CCCTGACAAGCGTTACCACA |
